# Gene Expression of Molecules Regulating Apoptotic Pathways in Glioblastoma Multiforme Treated with Umbilical Cord Stem Cell Conditioned Medium

**DOI:** 10.21315/mjms2019.26.6.4

**Published:** 2019-12-30

**Authors:** Novi Silvia Hardiany, Edward Christopher Yo, Eko Ngadiono, Septelia Inawati Wanandi

**Affiliations:** 1 Department of Biochemistry & Molecular Biology, Faculty of Medicine Universitas Indonesia, Jakarta, Indonesia; 2 Faculty of Medicine Universitas Indonesia, Jakarta, Indonesia

**Keywords:** glioblastoma multiforme, conditioned medium, umbilical cord blood stem cells, survivin, caspase, TRAIL

## Abstract

**Background:**

Glioblastoma multiforme (GBM) is the most malignant primary brain tumour and there is no definite cure. It has been suggested that there are significant interactions among mesenchymal stem cells (MSCs), their released factors and tumour cells that ultimately determine GBM’s growth pattern. This study aims to analyse the expression of molecules involved in GBM cell apoptotic pathways following treatment with the MSC secretome.

**Methods:**

A conditioned medium of umbilical cord-derived MSCs (UCMSC-CM) was generated by culturing the cells on serum-free αMEM for 24 h. Following this, human GBM T98G cells were treated with UCMSC-CM for 24 h. Quantitative reverse transcriptase-polymerase chain reaction (qRT-PCR) was then performed to measure the mRNA expression of survivin, caspase-9, TNF-related apoptosis-inducing ligand (TRAIL), DR4 and DcR1.

**Results:**

mRNA expression of caspase-9 in CM-treated T98G cells increased 1.6-fold (*P* = 0.017), whereas mRNA expression of survivin increased 3.5-fold (*P* = 0.002). On the other hand, TRAIL protein expression was upregulated (1.2-fold), whereas mRNA expression was downregulated (0.4-fold), in CM-treated cells. Moreover, there was an increase in the mRNA expression of both DR4 (3.5-fold) and DcR1 (1,368.5-fold) in CM-treated cells.

**Conclusion:**

The UCMSC-CM was able to regulate the expression of molecules involved in GBM cell apoptotic pathways. However, the expression of anti-apoptotic molecules was more upregulated than that of pro-apoptotic molecules.

## Introduction

Glioblastoma multiforme (GBM) is the most aggressive primary brain tumour and is most commonly found in humans (1). Due to its distinctive features and malignant behaviour, GBM is designated by World Health Organization (WHO) as a grade IV astrocytoma (2). The life expectancy of patients with GBM is usually less than a year (3). Despite advances in modern cancer treatments, its heterogeneous nature enables tumour cells to migrate over long distances into parts of the brain that are crucial for survival. The mean survival rate is only extended up to two or three months, a year at the most, after surgery, radiation therapy and chemotherapy (1).

A number of novel therapeutic approaches have been developed to combat tumour progression. During the era of precision medicine, researchers have begun to experiment with molecular variations in cancer, which include tumour-specific in vivo activation of apoptosis (4). Recently, the prospect of using mesenchymal stem cells (MSCs) for anti-cancer therapy has been extensively studied. MSCs can be genetically engineered to deliver antitumorigenic molecules into specific tumour sites in vivo (5). Because tumour development is largely orchestrated by inflammation, MSCs exhibit upregulation of various chemokine receptors (CCR1, CXCR5 and CCR2) and adhesion molecules (ALCAM, P-selectin and integrins) to facilitate extensive tropism towards specific tumour sites (5).

Recent studies have revealed that implanted MSCs only survive for a short period and have attributed their therapeutic potential largely to their secreted factors, known as the secretome (6). These bioactive factors include certain cytokines, growth factors, chemokines, microRNAs and other protein groups that regulate various physiological processes (6).

However, a growing body of literature has discovered that MSCs and their secretome can either support or suppress tumour development. The widely accepted notion is that MSCs influence cancer pathophysiology primarily via a paracrine mechanism, in which they produce biological factors that act on neighbouring cells. Through this paracrine mechanism, there are significant interactions among the stem cell, its released factors and the tumour cell that ultimately determine cancer progression. Just as stem cells affect cancer cells, the reverse is also true. Hence, the crosstalk between MSCs and cancer cells raises doubt regarding the clinical validity of MSCs as a potential treatment approach for GBM and other types of cancer (7).

Recently, de Castro et al. (8) found that the MSC secretome comprises proteins (IL6, NRP2, CCL2, etc.) involved in tumour growth and activates multiple pathways (VEGF, PDGF and Wnt pathways) that promote GBM cell growth, viability and migration. In line with their findings, our previous study also proved that a conditioned medium derived from umbilical cord mesenchymal stem cells (UCMSC-CM) increased the proliferation of the GBM T98G cell line and did not affect apoptosis observed using Annexin V analysis (9). This raises further questions as to whether UCMSC-CM affects the gene expression of molecules involved in GBM cell apoptotic pathways. Apoptosis itself is induced through activation of either intrinsic or extrinsic pathways regulated by pro-apoptotic and anti-apoptotic proteins (10–11).

One of the pro-apoptotic proteins involved in an intrinsic pathway is caspase-9. The activation of caspase-9 activates effector caspases, such as caspase-3 and caspase-7 (10). These caspases will later initiate cell apoptosis. However, caspase-9 can be blocked by survivin, which is an anti-apoptotic protein. Meanwhile, an extrinsic pathway is stimulated by TNF-related apoptosis-inducing ligand (TRAIL). TRAIL is expressed in MSCs and delivered into the tumour microenvironment. The ligand has also been found to be expressed in several tumour cell types, including GBM, although the dynamics of TRAIL protein expression and its physiological role in tumour cells remain unclear (11). The ability of TRAIL to selectively induce apoptosis depends on its ability to bind with TRAIL receptors. There are five known receptors of TRAIL, of which two induce apoptosis when cross-linked with the ligand (TRAIL-R1/DR4 and TRAIL-R2/DR5/KILLER). Three of them act as antagonistic decoy receptors (TRAIL-R3/DcR1, TRAIL-R4/DcR2 and osteoprotegerin) (11). The definitive binding between TRAIL and its agonistic/death receptors (DR4 and DR5) is crucial for activating the extrinsic apoptosis pathway (11). In contrast, binding the ligand with antagonistic/decoy receptors, such as DcR1, inhibits TRAIL signalling (11).

By investigating caspase-9, survivin, TRAIL, TRAIL agonistic receptor DR4 and TRAIL antagonistic receptor DcR1 expression in GBM cells after treatment with UCMSC-CM, the regulation of GBM cell apoptosis by UCMSC-CM can be elaborated. Therefore, this study aimed to measure the gene expression of caspase-9, survivin, TRAIL and its receptor to understand how UCMSC-CM affects apoptotic pathways in GBM cells.

## Materials and Methods

### GBM T98G Cell Culture

The human GBM T98G cell line (ATCC CRL-1690™) was obtained from Professor Alexander Brehm from the Institut fuer Molekularbiologieund Tumorforschung Phillipps Universitaet in Marburg, Germany. The T98G cell line (passage 20) was kept in a cryopreservation medium and stored in liquid nitrogen for long-term storage. The cell was cultured in high glucose Dulbecco’s Modified Eagle’s Medium (DMEM, Gibco) in T-25 culture flasks (Corning). The medium contained 10% foetal bovine serum (FBS, Biowest), 1% amphotericin B, 1% streptomycin-penicillin and sodium bicarbonate. It was conditioned at 37 °C in a humidified atmosphere of 95% O_2_ and 5% CO_2_, and replaced two times weekly. After the T98G cells reached 70%–80% confluency in the medium, they were sub-cultured (12).

### Generation of UCMSC-CM

Umbilical cord-derived MSCs (UCMSCs) were obtained from the umbilical cord of a mother who underwent a caesarean section after signing an informed consent document (13). Approval for the study was obtained from the Ethical Committee of the Faculty of Medicine at Universitas Indonesia. Isolated cells were characterised and tested for differentiation (13). The medium used in this study for growing 125,000 UCMSCs was minimum essential medium alpha (αMEM, Gibco) with 10% FBS (Biowest)/Glutamax (Gibco), 1% amphotericin and 1% streptomycin-penicillin in T-25 culture flasks (Corning). Cells were grown at 37 °C in a humidified atmosphere of 95% O_2_ and 5% CO_2_. Cells were collected from the medium after the culture reached 70%–80% confluence and they were then washed three times with 1x phosphate buffered saline (PBS). For the following 24 h, cells were grown in serum free-αMEM to prepare the UCMSC-CM. Thereafter, the samples were centrifuged to remove any cell debris and passed through a 0.22 μm filter. The resulting UCMSC-CM was then diluted with fresh high-glucose DMEM to achieve a final concentration of 50% (14).

### Induction of T98G Cells by UCMSC-CM

Our experimental setup was similar to that proposed by Hardiany et al. (14). About 400,000 T98G cells were plated in triplicate in 12-well plates in high-glucose DMEM with 10% FBS, 1% amphotericin B and 1% streptomycin-penicillin and were incubated overnight. On the following day, the medium of the T98G cells was replaced with 50% (v/v) UCMSC-CM and the culture was incubated for 24 h. The control group was set by culturing T98G cells in a medium containing a 1:1 ratio of DMEM and αMEM. The same experimental procedure was then repeated three times, yielding a total of nine samples.

### Analysis of Caspase-9, Survivin, TRAIL and TRAIL Receptor Expression

RNA samples from the treated T98G cells were collected using the Tripure RNA Isolation kit (Roche, Switzerland). The collected RNA was mixed with reagents from the SensiFAST™ SYBR® No-ROX KitBioline, UK), including reverse transcriptase enzyme, RNAse, NFW, Sensifast and primers (Table 1). qRT-PCR was performed on an Applied Biosystems 7500 Fast Instrument using standard procedures. The protocol for qRT-PCR is as follows: synthesis of cDNA was performed at 42 °C for 10 min. Then, inactivation using iScript reverse transcriptase was performed at 95 °C for 5 min, followed by 40 PCR cycles at 95 °C for 10 s. The annealing temperatures for the caspase-9, survivin, TRAIL, DR4 and DcR1 genes were 55 °C, 61.3 °C, 60 °C, 59 °C and 59 °C, respectively. Each procedure was performed for 30 s, followed by an extension temperature of 72 °C for 30 s. A melting curve analysis was performed at 95 °C for 1 min, followed by 55 °C for 1 min and 55 °C for 10 s, repeated for 80 cycles with a 0.5 °C increase every cycle. Non-template control was used as an internal control to ensure that no contamination occurred during the qRT-PCR process. PCR primers were designed from a sequence of each gene (GenBank^®^ NCBI) using the Primer3 programme. The list of primer sequences is shown in Table 1.

Relative gene expressions were calculated using the Livak method with the 18S rRNA gene as a reference gene. The formula for this method for relative mRNA expression is 2^−ΔΔCT^, in which CT is a cycle threshold representing an amplified qRT-PCR product. The ΔCT was obtained from the CT of the target gene both in CM-treated cells and in control cells after subtracting the CT of the reference gene, whereas the ΔΔCT was obtained from the ΔCT of the CM-treated cells after subtracting the ΔCT of the control cells.

### Analysis of ELISA TRAIL

The concentration of the TRAIL protein was measured in accordance with the assay procedure used with the Elabscience® human TRAIL enzyme-linked immunosorbent assay (ELISA) kit (sensitivity of 9.38 pg/mL with a detection range of 15.63 pg/mL–1,000 pg/mL). First, 100 μL of total protein was mixed with and added to the well of the micro ELISA plate, which had previously been coated with an antibody specific to TRAIL. The plate was covered and incubated at 37 °C for 90 min. The well was then emptied of all liquid, and 100 μL of Biotinylated Detection Ab working solution was added and mixed gently. The plate was then covered and incubated at 37 °C for 60 min. After the well was repeatedly washed using Wash Buffer (350 μL) and completely dried, 100 μL of Avidin-Horseradish Peroxidase (HRP) Conjugate working solution was added. The plate was then covered and incubated at 37 °C for 30 min, followed by 5 washes. Afterwards, 90 μL of substrate solution was added to the well, which was then sealed and incubated at 37 °C for 15 min. For the enzyme-substrate reaction to stop, 50 μL of stop solution (sulphuric acid solution) was added and converted the colour to yellow. The protein level of TRAIL was analysed by measuring the optical density (OD value) using a micro-plate reader set to 450 nm. The OD value was proportional to the protein concentration.

### Statistical Analysis

All data from the triplicate experiments are given as means and standard deviations (SD). Statistical analyses were performed using Student’s *t*-test and IBM’s SPSS Statistics Version 24 for Mac. The cut-off threshold selected for determining statistically significant differences was *P* < 0.05.

## Results

### Cell Morphology

**Figure 1:**
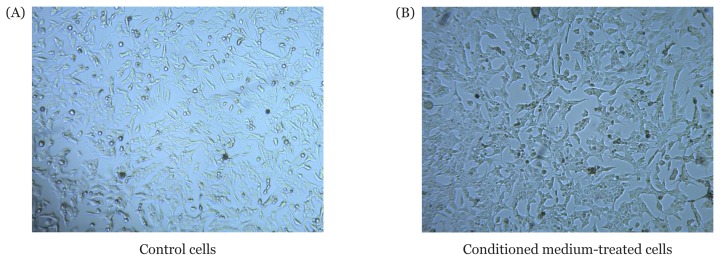
T98G cell morphology. 4×10^5^ cells were seeded in triplicate on a 12-well plate in high-glucose Dulbecco’s Modified Eagle’s Medium with 10% FBS, 1% amphotericin and 1% streptomycin-penicillin at 37 °C in 95% O_2_ and 5% CO_2_. The following day, the medium of treated cells was replaced with 50% (v/v) conditioned medium of UCMSC-CM, whereas the medium of the control cells was replaced with 50% (v/v) minimum essential medium alpha. Both cultures were further incubated for 24 h. Cell morphology was then observed using an inverted microscope (100× magnification)

Cell morphology was observed after 24 h of CM treatment. An inverted microscope (100× magnification) was used to observe and compare the morphology of the control T98G cells and CM-treated T98G cells. As observed using the microscope (Figure 1), the control and CM-treated T98G cells showed a fibroblast-like appearance, and both adhered to the plate. Moreover, the cell membrane was found to be intact, and the cell shape was unchanged. Hence, we concluded that there were no significant morphological differences between the control and CM-treated T98G cells.

### Caspase 9 and Survivin mRNA Expression in T98G Cells

To more closely examine the effects of UCMSC-CM on glioblastoma and the mechanisms by which UCMSC-CM induces apoptosis, we first examined caspase-9 and survivin mRNA levels in UCMSC-CM-treated T98G cells by qRT-PCR. As shown in Table 2, the expression of survivin mRNA was increased by approximately 3.5-fold (*P* = 0.002) in T98G cells treated with UCMSC-CM compared with T98G cells without UCMSC-CM treatment (controls). Similarly, caspase-9 mRNA expression showed a slight increase in UCMSC-CM-treated T98G cells, by approximately 1.6-fold (*P* = 0.017), compared with controls (Table 2).

### TRAIL and TRAIL Receptor mRNA Expression in T98G Cells

TRAIL mRNA expression was significantly downregulated (0.38-fold) in CM-treated T98G cells) in comparison to control cells (Table 2). In contrast, the expression of TRAIL protein was significantly higher in CM-treated T98G cells (38.1 ng/mL) than in control cells (32.6 ng/mL), as shown in Table 2. On the other hand, TRAIL DR4 expression was slightly upregulated (by 3.5-fold) (Table 2), whereas DcR1 expression was significantly upregulated (by 1368.5-fold) (Table 2), in CM-treated T98G cells compared to control cells.

## Discussion

### Intrinsic Apoptosis Pathway, Caspase-9 and Survivin

Survivin has been the focus of glioblastoma research due to its potent activities in regulating the cell cycle and regulating apoptosis through the inhibition of caspase activity (15–16). Survivin inhibits intrinsic apoptosis pathways by blocking the activity of caspase-9, an initiator caspase that plays a role in cell apoptosis (10). The activation of caspase-9 further activates effector caspases, such as caspase-3 and caspase-7 (10). These caspases initiate cell apoptosis. In addition to blocking caspase-9, survivin has the ability to block both caspase-3 and caspase-7 (8). Previous reports have shown that suppression of survivin induces apoptosis, slows proliferation and inhibits invasion by glioblastoma cells (17–18).

In the present study, we found that the mRNA expression of survivin increased by 3.5-fold in T98G cells treated with UCMSC-CM compared with controls. This observation is slightly at odds with those of previous studies, which showed that UCMSC-CM exhibited potent anti-glioblastoma activity, downregulated survivin expression and induced glioblastoma cell apoptosis (19). We also found that the mRNA level of pro-apoptotic caspase-9 expression increased by 1.6-fold in T98G cells treated with UCMSC-CM. These data show that the results of treatment of T98G cells with UCMSC-CM in this study may differ from those of previous studies due to differences in the cell lines used. The cytokines expressed and contained within UCMSC-CM appear to cause these events, although this study does not specifically measure how these changes may occur. Increased expression of caspase-9 has been suggested to be caused by multiple UCMSC-CM cytokines that in turn cause the cell to undergo stress conditions and eventually activate internal caspase pathways. However, more research must be conducted in order to prove this.

UCMSCs secrete factors that may cause or inhibit apoptosis in glioblastoma cells. Interleukin-6 (*IL-6*) is one of the multiple cytokines induced by UCMSCs (20). *IL-6* is a pro-inflammatory cytokine that induces both the growth and invasiveness of gliomas (21). A previous study showed that *IL-6* induces the mRNA expression of survivin in glioblastoma cells through mTORC2/NF-kB (22). As previously mentioned, survivin has the potential to block caspase-9 and promote the survival of glioblastoma cells (10, 17, 18). Despite the potential of survivin to suppress caspase-9, our results show that the mRNA expression of caspase-9 was slightly elevated in cells treated with UCMSC-CM. The transcriptional regulators of caspase-9 expression in glioblastoma cells have not yet been described. However, a previous study showed that internalisation of extracellular vesicles (EVs) secreted by UCMSCs into glioblastoma cells induced apoptosis (23). One potential mechanism is that the EVs released by UCMSCs contain a high number of microRNAs (miRNAs) with potential effects in regulating cellular molecular networks (24). miRNAs such as miR-106a and miR-448 were found within UCMSC’s EVs (US Patent 20190269739) (25). Both of these miRNAs induce apoptosis by increasing caspase-9 expression (26–27). Therefore, an intrinsic apoptosis pathway can be initiated through caspase-9 once EVs containing miRNAs are successfully internalised. Some studies have shown that miRNAs exhibit anti-apoptosis and growth inhibition properties (28–29).

Several previous studies have also shown conflicting outcomes regarding the effects of UCMSCs on glioblastoma survivability. Akimoto et al. (30) co-cultured UCMSCs together with glioblastoma cells and found that UCMSCs inhibited the growth of glioblastoma cells. In support of these findings, Bajetto et al. (20) found that cell-to-cell contact between UCMSCs and glioblastoma stem cells exhibited inhibitory effects on glioblastoma cell growth. However, soluble factors released by UCMSCs, such as cytokines, can exhibit stimulatory effects towards glioblastoma growth (20). Although the key molecules behind this phenomenon are not well known, the authors identified several cytokines that were released by UCMSCs, such as *IL-6*, *IL-8*, GRO and ENA-78 (20). Importantly, these cytokines promote the proliferation, migration and angiogenesis of glioblastoma cells (20). In another study, Yang et al. (19) treated glioblastoma U251 cells with UCMSC-CM showed that apoptosis was induced in the glioblastoma cells, with a significant downregulation of the survivin gene. This result is contradictory to those of Bajetto et al. (20). By using a different glioblastoma cell line (T98G) that had not been used by prior studies, this study provides information that supplements the study performed by Bajetto et al. (20), showing that UCMSC-CM increases the growth of glioblastoma cell lines (T98G), although there was a slight increase in the intrinsic apoptosis pathway.

### Extrinsic Apoptosis Pathway, TRAIL and Its Receptors

The binding of TRAIL with its agonistic/death receptors (DR4 and DR5) will initiate the extrinsic apoptosis pathway and prevent tumour proliferation. Death receptors possess a functional cytoplasmic death domain. Soon enough, adaptor protein Fas-associated death domain (FADD) and initiator caspase-8 and/ or -10 will be recruited to the death domain of DR4 or DR5, forming a death-inducing signalling complex (DISC). The formation of a DISC will activate effector caspases, such as caspase-3 and ultimately result in apoptosis or programmed cell death (4, 11).

First, our study revealed that the MSC secretome in the CM significantly upregulated TRAIL protein expression in tumour cells. This result is in accordance with findings from previous studies, which claimed that MSCs could be incorporated into the tumour microenvironment and regulate tumour progression and expression of factors (31–32).

This finding implies that MSCs secrete certain cytokines and transcription factors that may be responsible for increasing TRAIL gene transcription and subsequent protein expression in tumour cells. Previous investigations have revealed that several molecules have complementary binding sites in the TRAIL gene promoter and that they may alter its activity. One of these molecules is p53, which was proposed by Huang et al. (33) to be a crucial factor secreted by MSCs that can migrate to the tumour microenvironment. p53 is a very well characterised protein that is known for its tumour suppressing and DNA repair properties, especially when a cell is exposed to stress signals or mutagens (34). Interestingly, another study verified that p53 is capable of functioning as a transcription factor and elevates TRAIL promoter activity upon binding at the −630 position (35). This led us to hypothesise that p53 was present in the MSC secretome in CM and contributes to the upregulation of TRAIL expression in GBM cells. However, further investigation is necessary to study the exact mechanism of p53 uptake into tumour cells from the extracellular space and then into the nucleus. Alternatively, because this study did not focus on measuring the relative distribution of various factors in the MSC secretome, it is possible that the upregulation in TRAIL expression could be attributed to factors other than p53.

Contrary to what we expected, the expression of TRAIL mRNA was lower in the CM-treated T98G cells than that in the control cells. We initially assumed that the increase in the TRAIL protein levels in CM-treated T98G cells was due to an increase in mRNA expression as well. A possible explanation for this contradictory result is that a signalling gene like TRAIL tends to have unstable mRNA and proteins; consequently, there is a poor correlation between their expression (36). This lack of stability increases susceptibility to transcriptional and translational regulation, including inhibition of mRNA translation into proteins by miRNAs (36–37). Additionally, there is accumulating evidence that protein abundance is largely determined by varying posttranslational mechanisms and turnover rates. Also, when the protein level is already high, regulation via a negative feedback loop may be involved to inhibit further mRNA expression (36–38).

Our study also revealed that GBM cells expressed remarkably higher levels of DcR1 than DR4 upon treatment with the UCMSC secretome in CM. This result was surprising because an earlier study by Kim et al. showed that DR5— another death receptor similar in function to DR4—was significantly upregulated in glioma cells when the cells were cultured with MSC (39). This inconsistency may be due to the difference in the methods used; Kim et al. cultured glioma cells and MSCs together (direct interaction), whereas we extracted only the secreted factors of MSCs in the form of CM and induced the GBM cells with it (indirect interaction).

Interestingly, in correlation with our previous findings, Yoshida and Sakai (40) identified three p53-binding sites in the DR5 gene: +0.25 kb downstream of ATG, −0.82kb upstream of ATG and +1.25 kb downstream of ATG. It has been thought that p53 also modulates the transcription of the DcR1 gene, although its regulatory activity in the DcR1 gene is less understood compared to its regulatory activity in the DR4 and DR5 genes. Because we treated the GBM cells with MSC-secreted factors in CM instead of living cells, the predominant interaction may be of an indirect mode, such as paracrine signalling. In paracrine signalling, the p53 originates from the MSC secretome that migrated to the tumour microenvironment instead of from the tumour itself (33). The origin of p53, from the tumour’s intracellular or extracellular environment, may significantly affect which gene expressions are upregulated. Although direct cell-to-cell interaction, via the Notch signalling pathway, increases the expression of p53 in tumour cells and in turn upregulates the cells’ DR5 expression, it is possible that p53 originating from extracellular paracrine signalling is more likely to result in the upregulation of DcR1 rather than DR5.

The dual role of p53 in inducing both DR5 and DcR1 expression was believed to be a means of preserving the balance between cell apoptosis and cell survival (41). However, the DR4 gene’s mechanisms of regulation are poorly understood and therefore must be clarified. Although both DR4 and DR5 are TRAIL agonistic receptors capable of stimulating TRAIL-mediated apoptosis, Surget et al. (42) found that p53 regulated the expression of DR5 but had no effect on the expression of DR4.

DcR1 has been proposed to compete with DR4 and DR5 in binding with TRAIL (39). A high level of DcR1 may inhibit the signalling role of DR4 and DR5 in TRAIL-induced apoptosis (39). Decoy receptors (DcR1 and DcR2) do not possess a functional cytoplasmic death domain and interfere with the downstream apoptotic signal via distinct mechanisms (11, 43).

DcR1 is also able to activate cell survival signalling pathways via p38, extracellular signal-regulated kinases (ERK) and nuclear factor κ-light-chain-enhancer of activated B cells (NF-κB). This effect may contributed to a poor prognosis for GBM patients because it may further the tumour invasion into body tissues and organs (44). Further research should analyse the expression levels of every known TRAIL receptor type, as well as compare their levels among different cancer cell populations. In addition to mRNA expression measurement, protein expression should also be measured because mRNA expression may not be consistent with protein expression.

After reviewing the expression levels of the TRAIL protein and TRAIL receptors in GBM cells, we concluded that GBM cells treated with UCMSC-CM exhibited high expression of the TRAIL protein and the DcR1 receptor. Because neighbouring tumour cells can also interact with one another through paracrine signalling, there is a probability of TRAIL ligands from one GBM cell binding with DcR1 receptors from another GBM cell. The binding inhibits downstream apoptotic signalling and may lead to activation of the DcR1-associated survival signalling pathways instead. Hence, this may lead GBM cells to escape cell death and proliferate. However, the dynamics of TRAIL protein expression and its physiological role in tumour cells remain unclear and should be studied more deeply in the future.

This study should educate the public on the use of stem cells as a treatment for cancer, especially glioblastoma. There is still not enough information on how UCMSC-CM may induce or supress glioblastoma survivability genes. The use of UCMSC-CM as a glioblastoma treatment in the future should also be re-evaluated. Although there have been studies confirming its positive effects, conflicting studies (like ours) highlight the need for further in vitro research before proceeding to clinical trials.

## Conclusion and Future Directions

The clinical significance of this study relates to the use of UCMSC-CM for therapeutic approaches towards GBM. Further research should focus on quantifying the contents within UCMSC-CM, as they may contain UCMSC-EVs, miRNA and cytokines that regulate the growth of glioblastoma. In conclusion, our study revealed that the treatment of GBM cells with UCMSC-CM significantly increased the expression of survivin, TRAIL protein and DcR1, but it only slightly increased the expression of caspase-9 and DR4. These findings led us to propose that UCMSC-CM plays a significant role in regulating GBM cell apoptosis by controlling the expression of molecules involved in both intrinsic and extrinsic apoptosis pathways. The high expression of these anti-apoptotic molecules further supports the idea that UCMSC-CM is more likely to promote than inhibit tumour proliferation.

## Figures and Tables

**Table 1:** PCR primer sequences

Primer	Sequence
Caspase-9	Forward: TTG GTG ATG TCG AGC AGA AA
	Reverse: GGC AAA GTA GAT ATG GCG TC
Survivin	Forward: GCC AGA TGA CGA CCC CAT AGA GGA
	Reverse: TCG ATG GCA CGG CGC ACT TT
TRAIL	Forward: 5′-TGCGTGCTGATCGTGATCTT-3′
	Reverse: 5′-CTTGGAGTCTTTCTAACGAGCTG-3′
DR4	Forward: 5′-ACATGCAAGAGAGAAGATTCAGG-3′
	Reverse: 5′-TAACACCTAAGAGGAAACCTCTGG-3′
DcR1	Forward: 5′-CCAACGCTTCCAACAATGAA-3′
	Reverse: 5′-GGCATTGGCACCAAATTCTT-3′
18s rRNA	Forward: 5′-AAACGGCTACCACATCCAAG-3′
	Reverse: 5′-CCTCCAATGGATCCTCGTTA-3′

**Table 2:** A comparison of the expression of molecules regulating apoptotic pathways between the UCMSC-CM treated T98G and control groups

Variable	Mean (SD)		Mean differences (95% CI)	*t*-statistics (df)	*P*-value

	Control (*n* = 9)	Intervention (*n* = 9)			
Survivin mRNA	1.084 (0.466)	3.538 (1.905)	−2.45 (−3.73, −1.17)	−4.43 (8)	0.002
Caspase-9 mRNA	1.054 (0.352)	1.594 (0.686)	−0.54 (−0.95, −0.13)	−3.00 (8)	0.017
TRAIL mRNA	0.964 (0.085)	0.366 (0.024)	0.58 (0.521, 0,639)	22.847 (8)	0.038
TRAIL protein (ng/mL)	32.567 (0.583)	38.133 (0,469)	−5.567 (−6.325, −4.807)	−16.913 (8)	0.03
DR4 mRNA	0.890 (0.148)	3.090 (2.228)	2.20 (0.42, 3.98)	−2.86 (8)	0.021
DcR1 mRNA	1.029 (0.24)	1407.544 (572.153)	1406.52 (966.75, 1846.28)	−7.38 (8)	< 0.001

## References

[1] Holland EC (2000). Glioblastoma multiforme: the terminator. Proc Natl Acad Sci USA.

[2] Louis DN, Ohgaki H, Wiestler OD, Cavenee WK, Burger PC, Jouvet A (2007). The 2007 WHO classification of tumours of the central nervous system. Acta Neuropathol.

[3] Holmberg J, He X, Peredo I, Orrego A, Hesselager G, Ericsson C (2011). Activation of neural and pluripotent stem cell signatures correlates with increased malignancy in human glioma. PLoS One.

[4] Kuijlen JMA, Bremer E, Mooij JJA, den Dunnen WFA, Helfrich W (2010). Review: on TRAIL for malignant glioma therapy?. Neuropathol Appl Neurobiol.

[5] Ciavarella S, Dominici M, Dammacco F, Silvestris F (2011). Mesenchymal stem cells: a new promise in anticancer therapy. Stem Cells Dev.

[6] Vizoso FJ, Eiro N, Cid S, Schneider J, Perez-Fernandez R (2017). Mesenchymal stem cell secretome: Toward cell-free therapeutic strategies in regenerative medicine. Int J Mol Sci.

[7] Rhee K-J, Lee JI, Eom YW (2015). Mesenchymal stem cell-mediated effects of tumor support or suppression. Int J Mol Sci.

[8] Vieira de Castro J, Gomes ED, Granja S, Anjo SI, Baltazar F, Manadas B (2017). Impact of mesenchymal stem cells’ secretome on glioblastoma pathophysiology. J Transl Med.

[9] Hardiany NS, Yohana, Wanandi SI (2019). The impact of conditioned medium of umbilical cord-derived mesenchymal stem cells toward apoptosis and proliferation of glioblastoma multiforme cells. IOP Conf Ser: Earth Environ Sci.

[10] Chen X, Duan N, Zhang C, Zhang W (2016). Survivin and tumorigenesis: molecular mechanisms and therapeutic strategies. J Cancer.

[11] Hawkins CJ (2004). TRAIL and malignant glioma. Vitam Horm.

[12] Hardiany NS, Sadikin M, Siregar NC, Wanandi SI (2017). The suppression of manganese superoxide dismutase decreased the survival of human glioblastoma multiforme T98G cells. Med J Indones.

[13] Pawitan JA, Kispa T, Mediana D, Goei N, Fasha I, Liem IK (2015). Simple production method of umbilical cord derived mesenchymal stem cell using xeno-free materials for translational research. J Chem Pharm Res.

[14] Hardiany NS, Huang P, Dewi S, Paramita R, Wanandi SI (2018). Analysis of pluripotency marker expression in human glioblastoma multiforme cells treated with conditioned medium of umbilical cord-derived mesenchymal stem cells. F1000Research.

[15] Tang T-K, Chiu S-C, Lin C-W, Su M-J, Liao M-H (2015). Induction of survivin inhibition, G_2_/M cell cycle arrest and autophagic on cell death in human malignant glioblastoma cells. Chin J Physiol.

[16] Zhang H, Xu F, Xie T, Jin H, Shi L (2012). β-elemene induces glioma cell apoptosis by downregulating survivin and its interaction with hepatitis B X-interacting protein. Oncol Rep.

[17] Guo H, Wang Y, Song T, Xin T, Zheng Z, Zhong P (2015). Silencing of survivin using YM155 inhibits invasion and suppresses proliferation in glioma cells. Cell Biochem Biophys.

[18] Liu Y, Miao C, Wang Z, He X, Shen W (2012). Survivin small interfering RNA suppresses glioblastoma growth by inducing cellular apoptosis. Neural Regen Res.

[19] Yang C, Lei D, Ouyang W, Ren J, Li H, Hu J (2014). Conditioned media from human adipose tissue-derived mesenchymal stem cells and umbilical cord-derived mesenchymal stem cells efficiently induced the apoptosis and differentiation in human glioma cell lines in vitro. Biomed Res.

[20] Bajetto A, Pattarozzi A, Corsaro A, Barbieri F, Daga A, Bosio A (2017). Different effects of human umbilical cord mesenchymal stem cells on glioblastoma stem cells by direct cell interaction or via released soluble factors. Front Cell Neurosci.

[21] Shan Y, He X, Song W, Han D, Niu J, Wang J (2015). Role of *IL-6* in the invasiveness and prognosis of glioma. Int J Clin Exp Med.

[22] Zanca C, Villa GR, Benitez JA, Thorne AH, Koga T, D’Antonio M (2017). Glioblastoma cellular cross-talk converges on NF-κB to attenuate EGFR inhibitor sensitivity. Genes Dev.

[23] Del Fattore A, Luciano R, Saracino R, Battafarano G, Rizzo C, Pascucci L (2015). Differential effects of extracellular vesicles secreted by mesenchymal stem cells from different sources on glioblastoma cells. Expert Opin Biol Ther.

[24] Zou X-Y, Yu Y, Lin S, Zhong L, Sun J, Zhang G (2018). Comprehensive miRNA analysis of human umbilical cord-derived mesenchymal stromal cells and extracellular vesicles. Kidney Blood Press Res.

[25] Brodie C, Brodie S (2019). Mesenchymal stem cells populations, their products, and use thereof. United States patent.

[26] Huang Q, Ma Q (2018). MicroRNA-106a inhibits cell proliferation and induces apoptosis in colorectal cancer cells. Oncol Lett.

[27] Jin J, Wu Y, Zhou D, Sun Q, Wang W (2018). miR-448 targets Rab2B and is pivotal in the suppression of pancreatic cancer. Oncol Rep.

[28] Song H, Zhang Y, Liu N, Zhang D, Wan C, Zhao S (2016). Let-7b inhibits the malignant behavior of glioma cells and glioma stem-like cells via downregulation of E2F2. J Physiol Biochem.

[29] Mei J, Bachoo R, Zhang C-L (2011). MicroRNA-146a inhibits glioma development by targeting Notch1. Mol Cell Biol.

[30] Akimoto K, Kimura K, Nagano M, Takano S, To’a Salazar G, Yamashita T (2013). Umbilical cord blood-derived mesenchymal stem cells inhibit, but adipose tissue-derived mesenchymal stem cells promote, glioblastoma multiforme proliferation. Stem Cells Dev.

[31] Zhang Y, Daquinag A, Traktuev DO, Amaya-Manzanares F, Simmons PJ, March KL (2009). White adipose tissue cells are recruited by experimental tumors and promote cancer progression in mouse models. Cancer Res.

[32] Khakoo AY, Pati S, Anderson SA, Reid W, Elshal MF, Rovira II (2006). Human mesenchymal stem cells exert potent antitumorigenic effects in a model of kaposi’s sarcoma. J Exp Med.

[33] Huang Y, Yu P, Li W, Ren G, Roberts AI, Cao W (2014). p53 regulates mesenchymal stem cell-mediated tumor suppression in a tumor microenvironment through immune modulation. Oncogene.

[34] Solozobova V, Blattner C (2011). p53 in stem cells. World J Biol Chem.

[35] Kuribayashi K, Krigsfeld G, Wang W, Xu J, Mayes PA, Dicker DT (2008). TNFSF10 (TRAIL), a p53 target gene that mediates p53-dependent cell death. Cancer Biol Ther.

[36] Schwanhäusser B, Busse D, Li N, Dittmar G, Schuchhardt J, Wolf J (2011). Global quantification of mammalian gene expression control. Nature.

[37] Bartel DP (2009). MicroRNAs: target recognition and regulatory functions. Cell.

[38] Greenbaum D, Colangelo C, Williams K, Gerstein M (2003). Comparing protein abundance and mRNA expression levels on a genomic scale. Genome Biol.

[39] Kim SM, Lim JY, Park SI, Jeong CH, Oh JH, Jeong M (2008). Gene therapy using TRAIL-secreting human umbilical cord blood-derived mesenchymal stem cells against intracranial glioma. Cancer Res.

[40] Yoshida T, Sakai T (2004). Promoter of TRAIL-R2 gene. Vitam Horm.

[41] Ruiz de Almodóvar C, López-Rivas A, Redondo JM, Rodríguez A (2004). Transcriptional regulation of the TRAIL-R3 gene. Vitam Horm.

[42] Surget S, Chiron D, Gomez-Bougie P, Descamps G, Ménoret E, Bataille R (2012). Cell Death via DR5, but not DR4, is regulated by p53 in myeloma cells. Cancer Res.

[43] Mérino D, Lalaoui N, Morizot A, Schneider P, Solary E, Micheau O (2006). Differential inhibition of TRAIL-mediated DR5-DISC formation by decoy receptors 1 and 2. Mol Cell Biol.

[44] Thorburn A (2007). Tumor necrosis factor-related apoptosis-inducing ligand (TRAIL) pathway signaling. J Thorac Oncol.

